# The relationship between harsh parenting and adolescent depression

**DOI:** 10.1038/s41598-023-48138-w

**Published:** 2023-11-24

**Authors:** Mengge Li, Jirui Wang, Peng Ma, Wenyan Sun, Huoliang Gong, Yuan Gao

**Affiliations:** 1 School of Psychology, South China Normal University, Guangzhou, Guangdoong China; 2 School of Psychology, Henan University, Kaifeng, Henan China; 3 Children’s Hospital Affiliated of Zhengzhou University, Henan, China

**Keywords:** Psychology, Risk factors, Signs and symptoms

## Abstract

Guided by Beck’s cognitive model of depression, this study comprehensively explores the mechanisms linking harsh parenting, rumination, and victimization to the development of adolescent depression. A total of 5047 adolescents were assessed using the Harsh Parenting Scale, Rumination Scale, Olweus Bullying/Victimization Questionnaire, and Beck Depression Inventory. The results indicated that harsh parenting positively influences adolescent depression. Moreover, rumination emerged as an important mediator between harsh parenting and adolescent depression, similar to victimization. Additionally, we found that both rumination and victimization act as chain mediators in the relationship between harsh parenting and adolescent depression. These findings demonstrate that harsh parenting impacts adolescent depression mediated by rumination and victimization. By shedding light on these mechanisms, this study improves our comprehension of how harsh parenting influences adolescent depression and offers valuable insights for designing interventions to alleviate depression in this population.

## Introduction

Depression, as a major disease burden among adolescents^1^, poses various risks, including suicide, poor academic performance, substance abuse, and strained parent–child relationships^2–5^. The high rates of relapse and suicide associated with depression have made it a serious global public health issue in recent years^6^.

Based on Beck’s cognitive model of depression, depression arises from negative, pessimistic, and irrational patterns of thought. Specifically, under the influence of such cognitive patterns, individuals often hold negative views about their own abilities, worth, and attractiveness, which further exacerbates their depressive emotions^7^. It is speculated that harsh parenting may exert an impact on adolescent depression through mechanisms such as forming negative cognitive patterns and experiencing difficulties in social interactions. There have been numerous studies demonstrated that family risk factors, such as harsh parenting, and social risk factors, such as victimization, can significantly predict adolescent depression^8–10^. Additionally, several studies have provided empirical evidence for the mediating role of individual cognitive factors, such as rumination, in the association between harsh parenting, victimization experiences, and adolescent depression^12–14^. However, these studies often overlook the interactions between these risk factors. Some empirical evidence suggests that positive peer relationships can attenuate the link between harsh parenting and internalizing problems in adolescents^14^. Therefore, it is necessary to investigate the mediating role of victimization as a risk factor in the relationship between harsh parenting and adolescent depression. However, to our knowledge such an investigation has yet to be conducted. Thus, this study aims to comprehensively examine the impact mechanisms of both family and individual factors on adolescent depression by integrating harsh parenting, rumination, victimization, and adolescent depression into a chain-mediated model. Additionally, it aims to provide new research insights into reducing victimization and depression among adolescents.

Harsh parenting refers to a series of negative parenting behaviors, including physical aggression, verbal aggression, and compulsive/controlling behaviors, adopted by parents when they are dissatisfied with their children’s performance or when their children make mistakes^15^. These behaviors are often accompanied by parents’ negative emotions and attitudes towards their teenagers, such as apathy, anger, and insensitivity^16^. Beck’s cognitive model of depression emphasizes the importance of individual cognitive processes in the formation and onset of depression^7^. Multiple studies have shown a correlation between harsh parenting and negative cognitive patterns in adolescents, leading to the development of a negative coping style that focuses on processing threatening or negative information^8,17^. Therefore, harsh parenting may serve as a familial risk factor for adolescent depression. Specifically, children who experience harsh parenting may repeat scenes of abuse in their minds, further triggering depression symptoms such as anxiety, despair, and helplessness. Although many studies have shown a positive association between harsh parenting and childhood depression^18–20^. there is a lack of research exploring the mechanism of adolescent depression in the context of adverse family upbringing from a Chinese cultural perspective. We speculate that if harsh parenting practices affect depression in children from western cultural backgrounds, this relationship may also exist in Chinese adolescents. Based on these findings, hypothesis 1 is proposed: harsh parenting positively affects adolescent depression.

Rumination is defined as an individual’s tendency to persistently analyze negative events and experiences, focusing on their negative aspects, and experiencing repetitive and intrusive negative thoughts and emotions. This cognitive pattern has a significant negative impact on the lives of adolescents and may serve as a risk factor for depression^21,22^.According to Beck’s reciprocal-interaction model of depression, the quality of interactions with key individuals is correlated with the occurrence, duration, and recurrence of depression^7^. Given that their parents are the primary caregivers and influential figures in their lives, a harsh parenting style can convey negative self-related information to adolescents, such as “I am not worthy of love” and “I am worthless.” Consequently, adolescents may develop negative cognitive processing, exhibit a negative attribution style, engage in unconscious thinking, or develop a negative perception of the external world. They may interpret their pain in a negative light and repeatedly ruminate, ultimately resulting in rumination^23^. Prior research conducted by Gibb and Abela has also demonstrated that emotional abuse from parents can lead children to form negative cognitive reasoning styles, subsequently exacerbating symptoms of depression in children^24^. While there is a dearth of direct research on the specific correlations between harsh parenting, rumination, and adolescent depression, previous literature strongly suggests that harsh parenting can significantly influence the development of negative self-cognition in adolescents^9,25^, thereby amplifying their susceptibility to depression. Hence, this study proposes hypothesis 2: rumination mediates the relationship between harsh parenting and adolescent depression.

Olweus defines victimization as an individual being subjected to physical, verbal, or psychological attacks or intimidation by other individuals or groups, with characteristics of repetition, long-term duration, and an imbalance of power resulting in inner distress^26^. While previous research has not specifically examined the link between harsh parenting and adolescent depression through the mediation of peer victimization, the possibility of this relationship is suggested by social cognitive theory^27^, which emphasizes the perception and interpretation of adolescent social interactions and the impact of social environments on internalizing problems in adolescents. Dodge and Petitit’s research suggests that social cognitive deficits and hostile attribution biases are key factors contributing to adolescent aggression and emotional issues, and these cognitive biases may stem from negative experiences in the family environment and peer relationships^28–31^. Specifically, in families characterized by harsh parenting, adolescents may adopt a submissive and ingratiating attitude toward their parents to maintain their safety and psychological comfort and reduce the occurrence of harsh parenting^32,33^. These relationship patterns may transfer to their interactions with peers, leading to increased identification by bullies and an increased risk of victimization^34,35^. Barker found that teenagers who experience strict parenting styles tend to exhibit higher levels of social anxiety, withdrawal behaviors, and anger, which can result in more frequent peer conflicts and less effective coping strategies, increasing their vulnerability to victimization^36^. In addition, numerous studies have indicated that frequent experiences of being bullied can lead to more internalizing problems among adolescents, such as depression, feelings of loneliness, and anxiety^37,38^. Thus, this study proposes hypothesis 3: Being bullied mediates the relationship between harsh parenting and adolescent depression.

Recent studies have indicated that rumination may be a risk factor for adolescents experiencing peer victimization^39^. The resource allocation theory suggests that habitual rumination can deplete cognitive capacities that should otherwise be utilized for executive functioning (EFs), resulting in impairments in EFs. This impairment may lead to cognitive biases in judgment of one’s own and others’ behavior and intentions, interfering with the individual’s ability to adjust behaviors and cope effectively according to social demands^40^. Consequently, this may increase the risk of future peer victimization among adolescent individuals. In a 15-month longitudinal study of adolescents, it was found that those with higher levels of rumination exhibited impairments in EFs and were more likely to become targets of peer victimization in high school^41^. Additionally, a comprehensive analysis of multiple studies supported the association between rumination and victimization^42^, since adolescents who engage in rumination tend to focus on negative events, leading to more negative emotions and withdrawal behaviors in their interactions with peers. Such behaviors hinder friendly relationships with peers and make it difficult for adolescents to develop positive interpersonal connections. Moreover, these adolescents may have low expectations regarding problem-solving when faced with peer conflicts, which increases their vulnerability to victimization. Hence, this study proposes hypothesis 4: rumination and victimization mediate the relationship between harsh parenting and adolescent depression.

**Figure 1:**
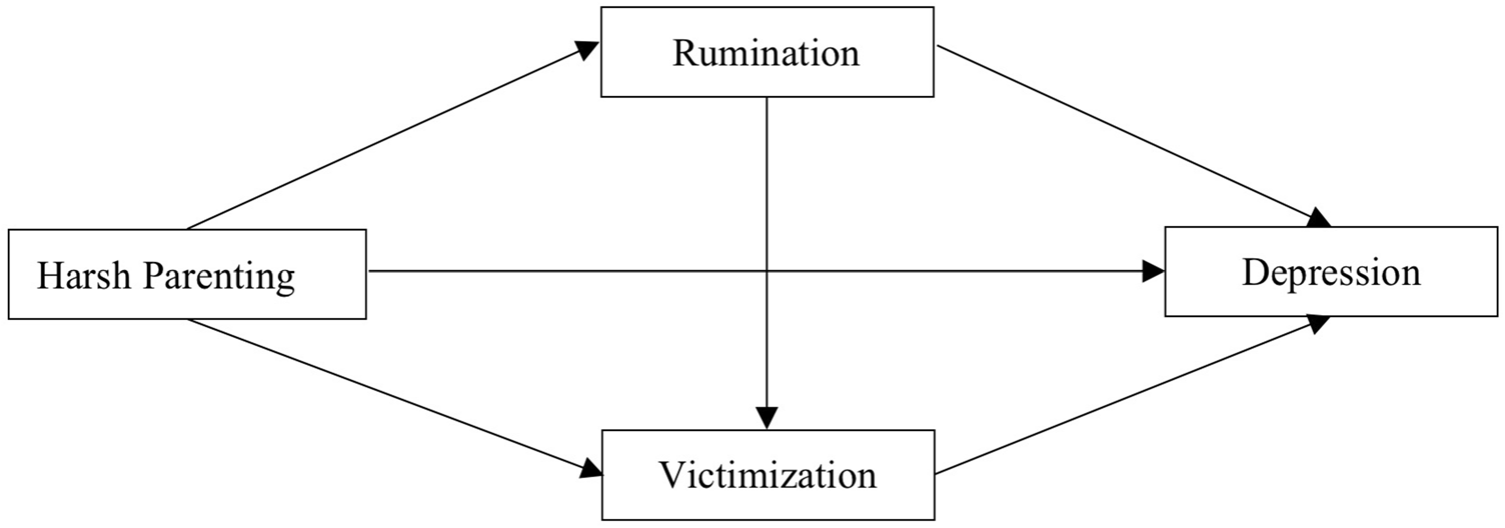
The moderated mediation model.

Overall, this study proposes a chain mediation model based on Beck’s cognitive model of depression to comprehensively examine the influence of family factors (harsh parenting), cognitive factors (rumination), and environmental factors (victimization) on adolescent depression. This integrated model provides a more comprehensive understanding of the development and manifestation of depression among Chinese adolescents. The findings of this study can be useful in identifying and providing support for adolescents who may be experiencing depressive symptoms and can contribute to the development of mental health education programs for Chinese adolescents. The hypothesized model for this study is shown in Fig. 1, illustrating the interrelationships between the variables.

## Research methods

### Participants

Participants were selected from Henan province in China. The valid sample included 5047 adolescents with female predominance (n = 2694/5047, 53.4%) and a mean age of 16.65 ± 1.22 (Range: 14–20 years). Among them, there were 2411 students in the first year of high school, 1238 in the second year, and 1398 in the third year. Additionally, 4025 participants came from rural areas, while 1022 were from urban areas.

### Procedure

A convenient cluster sampling method was used to conduct a survey and research at a high school in Henan province, central China. Approval was obtained from the school administration after clearly explaining our research plan. Subsequently, we informed the students and their parents about the nature of our tests in their respective classrooms. With the consent of both the students and their parents, we conducted assessments for students in grades 10–12 throughout the entire school. During the formal questionnaire survey, trained psychology graduate students and school teachers guided the teenagers to the school computer lab to complete the online survey. Before participating, all participants were informed of their right to withdraw from the study at any time. We also assured the participants about the privacy protection measures and emphasized the strictly academic use of the data with no dissemination to others. After providing clear instructions and explanations, the participants were asked to answer the questions based on their experiences. The participants were allowed to leave the computer lab after completing the survey. The data collection took place during the period between May and June of 2022, and it continued for one month.Finally, we used SPSS 26.0 for data entry, descriptive statistics, and correlation analysis. The data were subjected to the serial mediation model test using the ‘PROCESS’ macro.

The study involving human participants were reviewed and approved by Institutional Review Board of Henan Provincial Key Laboratory of Psychology and Behavior (Approval Number: 20220402003) and adhered to the principles of the Helsinki Declaration. Informed consent was obtained from all participants involved in the study and their guardians/parents.

### Measurements

#### Harsh parenting scale

The Harsh Parenting Scale, developed by Wang^43^, was used in this study. The scale comprises four items: “When I do something wrong, my father loses his temper with me and even shouts at me” and “When I do something wrong, he hits me with his hand or kicks me with his foot”. Participants rated the frequency of harsh parenting behavior on a 5-point Likert scale, with 1 point indicating “strongly disagree” and 5 points indicating “strongly agree.” Harsh parenting behaviors of fathers and mothers were assessed separately and then combined into a single indicator with higher scores indicating more frequent harsh parenting. The Cronbach’s α coefficient of the Harsh Parenting Scale in this study was 0.835.

#### Ruminative responses scale

The Chinese version of the Ruminative Response Scale (RRS) used in this study was translated and revised by Yang et al.^44^. The scale consists of 22 items categorized into three dimensions: depressive tendency, introspection, and obsessive thinking. Participants rated their level of agreement using a 4-point Likert-type scale, ranging from 1 (never) to 4 (always). Higher total scores indicate a higher level of rumination. The Cronbach’s α coefficient of the RRS in this study was 0.958, indicating a high level of internal consistency.

#### The olweus bully|victim questionnaire

The Chinese version of the Olweus Bully/Victim Questionnaire (OBVQ) used in this study was revised by Zhang et al.^45^. A subscale specifically measuring victimization was employed, consisting of a total of 6 questions that encompassed three dimensions: direct verbal bullying, indirect or relational bullying, and direct physical bullying. Participants were requested to rate the frequency of being bullied by their peers using a 5-point Likert scale (0 = none, 4 = several times a week). A higher score indicated a higher frequency of victimization experienced. The Cronbach’s α coefficient of this questionnaire in our study was 0.834.

#### Beck depression inventory

The Chinese version of the Beck Depression Inventory (BDI) used in this study was translated and revised by Yang et al.^46^. The scale consists of 21 items, each graded from 0 to 3. The total score of all items ranges from 0 to 63 points. According to the demarcation score standard provided by the scale, it can be divided into four degrees successively: a total score of 0–13 indicates no depressive tendency, a total score of 14–19 indicates mild depressive tendency, a total score of 20–28 indicates moderate depressive tendency, and a total score of 29–63 indicates severe depressive tendency. The Cronbach’s α coefficient of this scale in our study was 0.919.

## Results

### Common methods variance

Harman’s single-factor test was utilized in this study to assess and verify common method deviations. The unrotated factor analysis data identified seven factors with eigenvalues greater than 1 in this study. The total variance explained by the first factor was 33.61%. This value was lower than the critical value of 40%, suggesting minimal chance of significant common method bias in this study.

### Descriptive analysis and correlation analysis

Our results (Table 1) demonstrated that all variables were positively correlated (all *p* < 0.01). Notably, the correlation coefficients between Harsh Parenting and Rumination, Victimization, and Depression were 0.330, 0.316, and 0.386, respectively. The correlation between Rumination and Victimization and between Rumination and Depression was 0.313 and 0.687, respectively. Additionally, the correlation between Victimization and Depression was 0.362. These positive correlations among variables support the investigation of subsequent hypotheses. To address concerns about the high correlation between rumination and depression possibly being due to similar items in the two scales, additional correlation analyses were conducted between the three dimensions of rumination and depression and other variables. The results indicated that all three dimensions of the rumination scale exhibited a significant positive correlation with depression, not solely due to the high correlation between the rumination proneness subscale and the depression scale. Furthermore, gender and age were positively correlated with depression; however, the correlation coefficients were below 0.1, suggesting a weak relationship between gender, age, and depression and indicating that they may not have substantial importance or influence.

**Table 1:** Descriptive analysis and correlation matrix (n = 5047).

Index	M ± S.D	1	2	3	4	5	6	7	8	9
Gender	1.53 ± 0.499	1								
Age	16.65 ± 1.216	− 0.44**	1							
Harsh Parenting	11.762 ± 4.110	− 0.071**	− 0.078**	1						
Rumination	42.471 ± 13.024	0.014	0.013	0.330**	1					
Depressive Tendency	22.611 ± 7.539	0.016	0.001	0.342**	0.977**	1				
Introspection	10.424 ± 3.349	0.037**	0.002	0.299**	0.941**	0.879**	1			
Obsessive thinking	9.435 ± 2.997	− 0.018	0.050**	0.259**	0.895**	0.808**	0.812**	1		
Victimization	6.984 ± 2.487	− 0.116**	0.006	0.316**	0.313**	0.314**	0.272**	0.286**	1	
Depression	12.123 ± 9.441	0.077**	− 0.066**	0.385**	0.687**	0.712**	0.621**	0.543**	0.362**	1

*p < 0.05, **p < 0.01, ***p < 0.001; the same below.

### Chain mediation effect analysis

All variables were normalized before formal analysis. In order to control the influence of other factors, gender and age were treated as covariables. Model 6 has been used to analysis chain mediation effect in process 3.0.

The results showed that the fitting of all models are up to standard (see Table 2). From the model effect, the effect of Harsh Parenting on Rumination is significant (coeff = 0.307, t = 22.862, *p* < 0.001).

**Table 2:** Chain mediating effect analysis (n = 5047).

Model					Coeff	
DV	IV	*F*	*R*	*R*^2^	*β*	*t*
Rumination	Gender	159.371	0.370	0.137	0.037	2.841**
	Age				0.067	3.030**
	Grade				− 0.025	− 1.137
	Economic situation				− 0.159	− 11.911***
	Harsh Parenting				0.307	22.862***
Victimization	Gender	163.629	0.404	0.163	− 0.100	− 7.703***
	Age				0.077	3.545***
	Grade				− 0.073	− 3.368***
	Economic situation				− 0.036	− 2.716**
	Harsh Parenting				0.225	16.186***
	Rumination				0.231	16.657***
Depression	Gender	825.698	0.731	0.534	0.091	9.378***
	Age				− 0.033	− 2.035*
	Grade				− 0.027	− 1.664
	Economic situation				− 0.089	− 8.983***
	Harsh Parenting				0.136	12.775***
	Rumination				0.580	54.542***
	Victimization				0.136	12.953***

DV: the outcome variable, IV: the predictor variable; n = 5047.

The positive effect of Harsh Parenting on Victimization is significant (coeff = 0.225, t = 16.186, *p* < 0.001), and the Rumination has a positive significant effect on Victimization (coeff = 0.231, t = 16.657, *p* < 0.001); Furthermore, the positive effect of Harsh Parenting on Depression is significant (coeff = 0.136, t = 12.775, *p* < 0.001), the Rumination has a positive significant effect on Depression (coeff = 0.580, t = 54.542, *p* < 0.001), the positive effect of Victimization on Depression is significant (coeff = 0.136, t = 12.953, *p* < 0.001).

Further mediation analysis revealed that rumination and victimization have a significant chain mediation effect between harsh parenting and depression, suggesting that harsh parenting not only directly influences depression but also indirectly influences depression through the chain mediation role of rumination and victimization.

Specifically, the direct effect (0.136) and the total mediation effect (0.218) accounted for 38.418% and 61.582% of the total effect (0.354), respectively (see Table 3). Among the total mediation effect (0.218), the mediation effects of Rumination (0.178), Victimization (0.031), and their chain (0.010) accounted for 50.282%, 8.757%, and 2.713% of the total effect, respectively.

**Table 3:** Total effect, direct effect and the mediating effect.

	Effect	S.E	t	Lower 2.5%	Upper 2.5%	Percentage (%)
Direct effect	0.136	0.011	12.775***	0.115	0.156	38.418
Indirect effect	0.218	0.013	16.769***	0.193	0.244	61.582
M1	0.178	0.010	17.282***	0.158	0.199	50.282
M2	0.031	0.015	4.857**	0.019	0.044	8.757
M3	0.010	0.002	5.647**	0.006	0.013	2.713
Total effect	0.354	0.013	27.324***	0.328	0.379	

**Figure 2:**
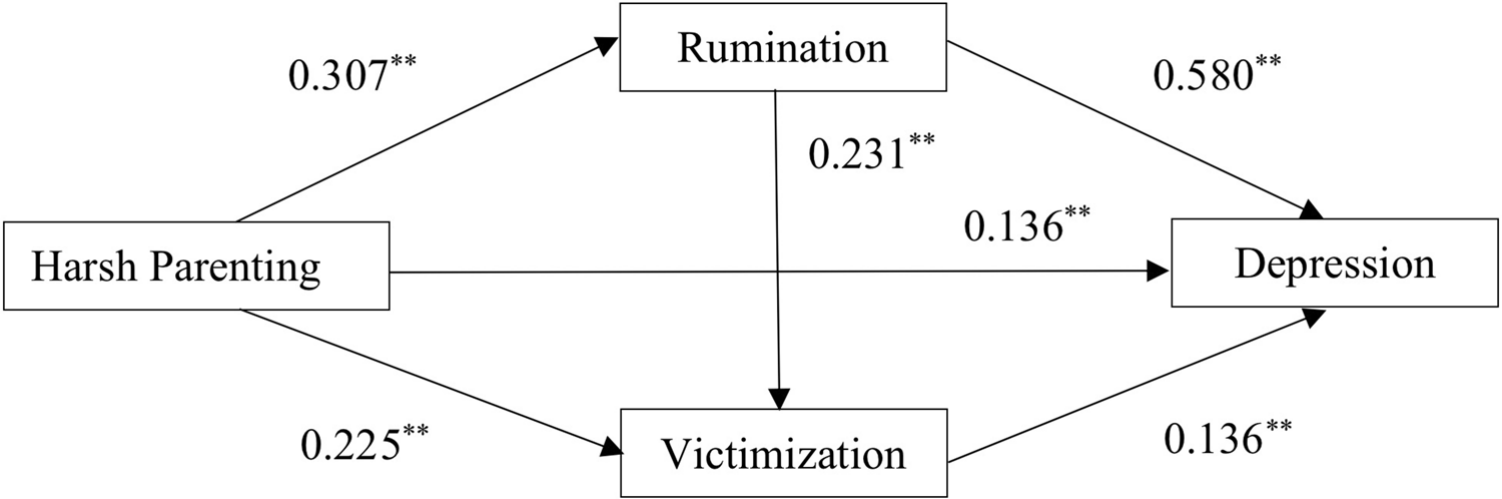
Schematic diagram of model.

The path coefficients of the chain mediation mode are presented in Fig. 2. M1: Harsh Parenting → Rumination → Depression; M2: Harsh Parenting → Victimization → Depression; M3: Harsh Parenting → Rumination → Victimization → Depression. The lower 2.5% and upper 2.5% refer to the interval estimates of Bootstrap 2.5% and 97.5%.

## Discussion

The results of this study indicate a significant correlation between adolescent gender and depression (r = 0.07), as well as between age and depression (r = 0.06) (*p* < 0.01). The corresponding coefficients of determination were 0.049 and 0.036, respectively. Specifically, the statistical significance of these two variables suggests that the influence of gender and age on adolescent depression may be genuinely present rather than driven by random factors. This also suggests the existence of a stable correlation between gender, age, and adolescent depression. However, it is important to note that although these two variables demonstrate statistical significance, their explanatory power for the overall condition of adolescent depression remains relatively low. This indicates that there may be other unconsidered factors that have a more significant impact on adolescent depression. Several factors may have contributed to these findings. Firstly, our study focused on a sample of high school students, where both male and female students share similar living environments and academic pressures. The primary stressors they encounter primarily stem from academic demands. Consequently, the correlation between gender and depression may be relatively small, which aligns with previous studies conducted in China^47^. Furthermore, due to the exclusive inclusion of high school students in our sample, the limited age range might contribute to the reduced correlation between age and depression. It is highly conceivable that the cross-sectional design employed in this study could not capture the variations in depressive symptoms across different age groups. Longitudinal or follow-up studies have the potential to provide more accurate observations regarding age-related differences in depression.

Our findings provide support for hypothesis 1 of this study, indicating that harsh parenting positively impacts adolescent depression^48^, consistent with Beck's cognitive model of depression^49^, which suggests that parents who adopt critical, angry, and hostile parenting styles can communicate a message of excessive flaws to their adolescents, conducive to the development of a negative self-cognitive schema, causing negative self-experiences such as loneliness, low self-esteem, and self-abandonment, which significantly increases the risk of depression among adolescents^49^. Additionally, despite the Beck’s cognitive model of depression originating from a Western cultural context, this study found no significant differences between Eastern and Western cultures. In the cultural context of China, parents may employ more harsh parenting behaviors under the belief that they aid in cultivating positive habits and have a beneficial impact on their children^50^. However, these harsh parenting practices also contribute to the self-cognition of Chinese adolescents, resulting in an increased risk of depression. This finding aligns with previous cutting-edge research conducted on Chinese adolescents^14,51^.

Our results also support the Family Systems Theory, indicating that negative parental caregiving behaviors, such as indifference, punishment, and excessive control, can disrupt the supportive atmosphere within the family, leading to increased stress and negative experiences for adolescents. These adverse family environments may contribute to a sense of inability to cope with the pressures and setbacks of daily life, thereby increasing the risk of depression. In conclusion, it can be inferred that harsh parenting positively impacts adolescent depression.

The results support hypothesis 2 of this study, indicating that rumination plays a mediating role between harsh parenting and adolescent depression. The experience of a series of negative life events resulting from harsh parenting can create a gap between an individual’s reality and expectations, leading to repetitive reflection on the causes and consequences of negative events, ultimately resulting in a state of rumination. For example, individuals who frequently experience criticism or verbal abuse from their parents may repeatedly contemplate their own shortcomings or mistakes, often attributing problems to themselves within a negative emotional context, thereby increasing their risk of psychological disorders^52^. Generally, adolescents who experience harsh parenting are more likely to develop negative cognitive patterns, fall into negative thinking habits, and ruminate on the reasons and outcomes of problems, leading to a weakened ability to understand others, excessively high expectations of people and things, and a heightened sense of disappointment in life, ultimately increasing their risk of depression. Therefore, rumination serves as a mediator in the relationship between harsh parenting and adolescent depression.

The results of this study support hypothesis 3, indicating that victimization plays a mediating role between harsh parenting and adolescent depression. Adolescents who grow up with warm and affectionate parenting styles are more likely to develop mature emotional regulation and adhere to normative moral standards, leading to healthier peer relationships and lower rates of victimization^53^. Specifically, harsh parenting may blur appropriate behaviors and boundaries in social interactions, leading individuals to lack positive strategies for dealing with provocation and conflict. The learned pattern of behavior may result in passive or negative responses when facing victimization, preventing adolescents from developing effective problem-solving strategies. Consequently, such individuals often exhibit poor social skills, emotional understanding, and regulation abilities, experiencing higher rates of victimization^54^. Overall, individuals who experience harsh parenting tend to rely on fixed thinking patterns and adopt passive coping strategies when facing challenging social relationships. The lack of emotional support and guidance from parents contributes to the presence of negative emotions towards oneself and others, further exacerbating the risk of depression.

The results of this study support hypothesis 4, indicating that rumination and victimization play a chain mediating role between harsh parenting and adolescent depression. This finding aligns with the resource allocation theory and suggests that rumination makes individuals excessively sensitive in their evaluations and perceptions of others, potentially influencing their coping strategies and resulting in misguided responses to others’ behavior and intentions. Consequently, their acceptance and support among peers are impacted, rendering them more susceptible to attacks and harm.

In summary, harsh parenting transmits negative messages to adolescents, subsequently internalizing and integrating into their self-concept, forming a negative self-image^55^. Additionally, within the framework of rumination, individuals struggle to acquire effective emotional regulation strategies and develop appropriate coping mechanisms for peer interactions. Consequently, they face difficulties in managing social pressures and become more easily targeted for bullying, leading to inappropriate emotional reactions and overall heightened risk of developing depression.

## Conclusions

This study examines the relationship between parental harsh parenting, rumination, victimization, and adolescent depression based on the Family Systems Theory and Beck’s cognitive theory of depression. The research findings indicate that parental harsh parenting has a positive impact on adolescent depression. Rumination and victimization play a mediating role in the link between parental harsh parenting and adolescent depression. To reduce the occurrence of adolescent depression, parents should decrease their use of harsh parenting and promote the psychological well-being of their adolescents.

### Strengths and limitations

Our study has several notable strengths. Firstly, the findings of this study highlight the significant impact of harsh parenting on adolescent depression, thereby validating the applicability of the Family Systems Theory within the Chinese context. These results contribute to the further enrichment of the Family Systems Theory, enhancing its relevance and content across diverse cultural backgrounds. Secondly, the study reveals that harsh parenting not only directly affects adolescent depression but also amplifies the occurrence of victimization by influencing adolescents' rumination, thereby increasing their vulnerability to depression. This study presents novel research perspectives for investigating the underlying mechanisms of adolescent depression, providing valuable insights for future researchers in this field. Lastly, this research offers fresh insights for psychological counseling practices aimed at mitigating adolescent depression. Counselors can foster effective communication with parents, encouraging them to reduce harsh parenting behaviors and ultimately alleviating adolescent depression symptoms while fostering their overall psychological well-being.

However, there are still some limitations and shortcomings that should be addressed in future research. Firstly, this study only selected Chinese adolescents for research. To increase the contrast in cultural differences, future research could consider cross-national and cross-cultural studies. Secondly, this study only measured the effects of parents’ harsh parenting during adolescence on depression through rumination and victimization but did not explore the impact of harsh parenting on the maintenance and recurrence of depression in adolescents. In the future, researchers can use longitudinal designs to more clearly explain how parental harsh parenting influences the development, maintenance, and recurrence of adolescent depression. Finally, this study only explored the impact mechanism of adolescent depression from the family level and did not conduct a comprehensive analysis and exploration from the perspective of schools.

In conclusion, future research should combine schools and families to explore the formation mechanism of adolescent depression from the perspective of family-school cooperation.

### Ethical approval

Ethics Statement The studies involving human participants were reviewed and approved by the Research Ethics Committee of the Institute of Psychology and Behavior of author’s university. Written informed consent to participate in this study was provided by the participants’ legal guardians/next of kin.

### Informed consent

Informed consent was obtained from all individual participants included in the study.

## Data Availability

The datasets generated during the current study are not publicly available due [The schools of the participants and the institutions from which the raw data came disagreed about making the data public] but are available from the corresponding author on reasonable request.
